# Synthetic Imidazopyridine-Based Derivatives as Potential Inhibitors against Multi-Drug Resistant Bacterial Infections: A Review

**DOI:** 10.3390/antibiotics11121680

**Published:** 2022-11-22

**Authors:** Bharat Kumar Reddy Sanapalli, Akram Ashames, Dilep Kumar Sigalapalli, Afzal B. Shaik, Richie R. Bhandare, Vidyasrilekha Yele

**Affiliations:** 1 Department of Pharmacology, NIMS Institute of Pharmacy, NIMS University Rajasthan, Jaipur 303121, Rajasthan, India; 2 College of Pharmacy & Health Sciences, Ajman University, Ajman P.O. Box 340, United Arab Emirates; 3 Center of Medical and Bio-Allied Health Sciences Research, Ajman University, Ajman P.O. Box 340, United Arab Emirates; 4 Department of Pharmaceutical Chemistry, Vignan Pharmacy College, Jawaharlal Nehru Technological University, Vadlamudi 522213, Andhra Pradesh, India; 5 St. Mary’s College of Pharmacy, St. Mary’s Group of Institutions Guntur, Jawaharlal Nehru Technological University Kakinada, Guntur 522212, Andhra Pradesh, India; 6 Department of Pharmaceutical Chemistry, NIMS Institute of Pharmacy, NIMS University Rajasthan, Jaipur 303121, Rajasthan, India

**Keywords:** imidazopyridine, synthetic approaches, antibacterial activity, multi-drug resistance, SAR studies

## Abstract

Fused pyridines are reported to display various pharmacological activities, such as antipyretic, analgesic, antiprotozoal, antibacterial, antitumor, antifungal, anti-inflammatory, and antiapoptotic. They are widely used in the field of medicinal chemistry. Imidazopyridines (IZPs) are crucial classes of fused heterocycles that are expansively reported on in the literature. Evidence suggests that IZPs, as fused scaffolds, possess more diverse profiles than individual imidazole and pyridine moieties. Bacterial infections and antibacterial resistance are ever-growing risks in the 21st century. Only one IZP, i.e., rifaximin, is available on the market as an antibiotic. In this review, the authors highlight strategies for preparing other IZPs. A particular focus is on the antibacterial profile and structure–activity relationship (SAR) of various synthesized IZP derivatives. This research provides a foundation for the tuning of available compounds to create novel, potent antibacterial agents with fewer side effects.

## 1. Introduction

Fused pyridines are an outstanding class of heterocycles with a diverse pharmacological profile which researchers have explored extensively [1,2,3,4,5]. Among them, imidazopyridine (IZP), i.e., imidazole fused with pyridine ring, comprises an important class of pharmacologically active nitrogen-containing fused heterocycles [6,7,8,9]. All these derivatives exhibit a wide range of pharmacological activities, such as antipyretic, analgesic, anxiolytic, antiprotozoal, antibacterial, antitumor, antifungal, anti-inflammatory, and antiapoptotic activities [10,11,12,13,14,15]. Moreover, several drugs which are available on the market, like necopidem and saripidem (anti-anxiolytic) [16], olprinone (acute heart failure) [17], zolpidem (insomnia) [18], zolimidine (peptic ulcer) [19], alpidem (anxiolytic) [18], and rifaximin (antibiotic) [20] possess IZP moieties.

**Figure 1:**
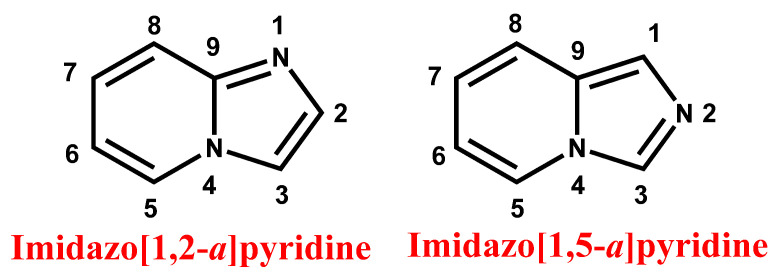
General structure and nomenclature of imidazopyridine.

Consequently, there is a continuous effort to conceive novel strategies to develop imidazopyridine with different substituents at various positions on the imidazole and pyridine moieties (Figure 1) and to evaluate the biological activities of the resulting compounds. In this review, we concisely present the important synthetic methodologies of imidazopyridines reported to date, along with their antibacterial activities against wild and resistant pathogens.

## 2. Strategies for the Synthesis of Imidazopyridines

**Figure 2:**
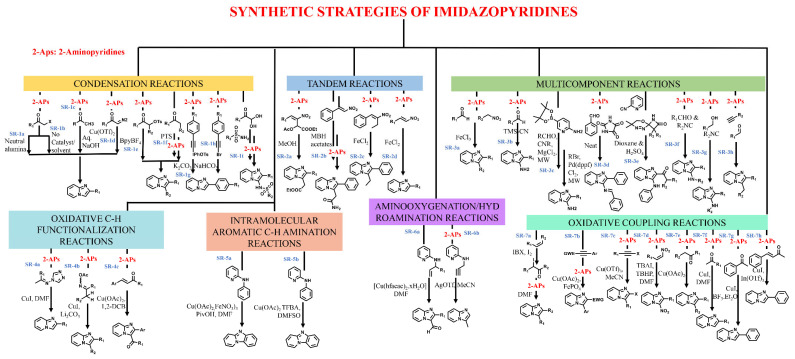
Synthetic strategies for the preparation of imidazopyridines.

Several approaches, e.g., multicomponent, condensation, oxidative coupling, amino-oxygenation, tandem reaction, hydroamination, etc., have been put forward for synthesizing this privileged scaffold. These approaches have been consequently optimized to produce IZP and its derivatives with high yield and purity. The literature describes some important synthetic routes, which are summarized in Figure 2. 

The (SR)-1a synthetic route is a traditional strategy involving a simple condensation reaction of 2-aminopyridines with α-haloketone in the presence of neutral alumina, as proposed by Sahu et al., 2005 [21]. Zhu et al., 2009 [22], later optimized this process. They reported the synthesis (SR-1b) of IZP with α-haloketone and 2-aminopyridine in the absence of a catalyst and in solvent-free conditions at 60 °C. Stasyuk et al., 2012 [23], reported the synthesis (SR-1c) of IZP from ketones via the in situ production of α-iodoketone. A library of compounds was synthesized via Ortoleva-King reaction followed by ring closure. The authors then studied the optoelectronic properties of these compounds. IZP with 2-hydroxyphenyl at position 2 established an excited-state intramolecular proton transfer (ESIPT), displaying strong emission bands in the blue region.

Further, α-diazoketone is just as crucial as α-haloketone for synthesizing IZPs (SR-1d), as reported by Yadav et al., 2007 [24]. IZPs were obtained with good selectivity and yields from the 2-aminopyridines and aliphatic/aromatic α-diazoketone in the presence of Cu(OTf)_2_. This reaction mechanism involved imine formation and nitrogen insertion as critical synthetic steps. Xie et al., 2004 [25], synthesized IZPs from the reaction of α-tosyloxyketones with 2-aminopyridines in the presence of an ionic liquid, i.e., BPyBF4 (SR-1e). In general, the use of organic solvents as a reaction medium requires much time and controlled temperature. To avoid these limitations, ionic solvents have been applied.

Ueno and Tang, 2004 [26], synthesized IZPs from alcohol and ketone via the in situ generation of α-sulfonyloxyketones (oxidative conversion) in the presence of macroporous polystyrene sulfonic acid and (diacetoxyiodo)benzene in the first step. The second step was carried out with 2-aminopyridine in the presence of potassium carbonate and acetonitrile (SR-1f). Liu et al., 2004 [27], developed a robust and simple synthetic method for the synthesis of IZPs from alkynyl(phenyl)iodonium salts with 2-aminopyridine in the presence of potassium carbonate and chloroform via [3,3]-sigmatropic rearrangement followed by intramolecular cyclization (SR-1g). In addition, Wu et al., 2011 [28], synthesized 3-arylIZPs with 1-bromo-2-pheylacetylenes/1,1-dibromo-2-phenylethenes and 2-aminopyridines in the presence of catalyst-free cascade reaction (SR-1h) and utilized only sodium bicarbonate as a base for the transformation. Yu et al., 2014 [29], reported a facile one-pot synthesis of N-(imidazo [1,2-a]pyridin-3-yl)sulfonamides using 2-aminopyridines, sulfonamides, and arylglyoxal hydrates in the presence of zinc chloride (SR-1i).

Tandem reactions have also been employed for the synthesis of IZPs. Nair et al., 2021 [30], synthesized IZPs from a 2-aminopyridine reaction with Morita-Baylis-Hillman (MBH) acetates of nitroalkenes in methanol. This reaction involved Michael’s addition of 2-aminopyridine with MBH acetates (SR-2a & 2b). Further, Yan et al., 2012 [31], synthesized 3-methyl-2-arylimidazo [1,2-a]pyridine derivatives via Fe(II)-catalyzed tandem coupling of 2-methylnitroolefins and 2-aminopyridines (SR-2c). Santra et al., 2013 [32], also developed a facile synthesis method for 3-unsubstituted IZPs from nitroolefins and 2-aminopyridine in the presence of ferric chloride (SR-2d).

Nevertheless, employing a multi-component strategy has also been found to be effective for synthesizing IZP and its derivatives. Yan et al., 2014 [33], described a one-pot, three-component approach for synthesizing IZPs using aldehyde, nitroalkane, and 2-aminopyridine in the presence of iron as a catalyst (SR-3a). Further, Schwerkoske et al., 2005 [34], used a three-component approach for IZP via the reaction of trimethylsilyl cyanide, aldehyde, and 2-aminopyridine in the presence of scandium triflate under microwave irradiation (SR-3b). DiMauro et al., 2007 [35], developed a multi-component microwave-assisted one-pot cyclization/Suzuki coupling reaction to synthesize 2,6-disubstituted-3-amino-IZPs from isonitriles, aldehydes, bromo derivatives, and 2-aminopyridines (SR-3c).

Another study by Adib et al., 2008 [36], established an efficient multi-component synthesis method for 3-amino-2-aryl IZPs from imidazolidine-2,4,5-trione, 2-aminopyridines and benzaldehydes under solvent-free conditions (SR-3d). Shao et al., 2011 [37], reported a one-pot reaction of β-lactam carbenes with 2-pyridyl isonitriles followed by acidic hydrolysis in 1,4-dioxane, resulting in 2-carbonyl-3(pyridylamino)imidazo-[1,2-a]pyridines with good quality and high yield (SR-3e).

In addition, Khan et al., 2012 [38], proposed the Ugi reaction for the synthesis of IZPs, employing aromatic aldehyde, aromatic amidine, and isocyanide in the presence of bromodimethylsulfonium bromide as a catalyst at room temperature (SR-3f). Ramesha et al., 2013 [39], developed an efficient synthesis method of IZPs from a variety of alcohols using polyphosphonic anhydride (SR-3g) as a catalyst. Another copper-catalyzed multi-component approach was followed by Chernyak et al., 2010 [40], for synthesizing IZP from 2-aminopyridines, aldehydes, and terminal alkynes (SR-3h).

An oxidative C-H functionalization strategy was also utilized for the synthesis of IZPs from N-(alkylidene)-4H-1,2,4-triazole-4-amines and pyridines in the presence of a copper catalyst in DMF, as reported by Yu et al., 2013 (SR-4a) [41]. A later study by Huang et al., 2013 [42], synthesized IZPs through the oxidative cyclization of pyridines with ketone oxime esters in the presence of copper-iodide as a catalyst (SR-4b). Another methodology for the generation of 3-aroyl IZPs through oxidative coupling between 2-aminopyridines and chalcones in the presence of copper as catalyst (SR-4c) was reported by Monir et al., 2014 [43].

The literature also proposes the synthesis of fused IZPs through intramolecular aromatic C-H amination. Wang et al., 2010 [44], synthesized pyrido [1,2-a]benzimidazole in the presence of copper and iron catalysts through direct intramolecular aromatic C-H amination (SR-5a). A study by Masters et al., 2011 [45], described the facile synthesis of pyrido [1,2-a]benzimidazole via copper-catalyzed amination (SR-5b).

Aminooxygenation and hydroamination are the other two effective strategies for the synthesis of IZPs. Wang et al., 2011 [46], developed an elegant intramolecular dehydrogenative aminooxygenation reaction for the synthesis of IZPs comprising a formyl group from the acyclic precursors in the presence of copper in DMF (SR-6a). Chioua et al., 2013 [47] developed a regioselective synthesis technique for 3-methyl IZPs through silver-mediated cycloisomerization of N-(prop-2-yn-1-yl)pyridine-2-amines using acetonitrile as a solvent (SR-6b).

Another strategy, i.e., oxidative coupling, was proposed by Donohoe et al., 2012 (SR-7a) [48] for the synthesis of IZPs through the formation of in situ α-iodoketones which react with available alkenes and 2-aminopyridine. Further, Zeng et al., 2012 [49], reported a facile and robust synthesis method for IZPs through an oxidative coupling reaction of alkyne with aminopyridine in the presence of copper/iron catalysts (SR-7b). A study by Gao et al., 2013 [50], described a one-pot oxidative coupling methodology for the generation of 2-haloimidazo [1,2-a]pyridines from 2-aminopyridines and haloalkynes in the presence of copper triflate (SR-7c).

The oxidative coupling reaction uses 2-aminopyridine and nitroalkenes, which are good Michael acceptors, for the synthesis of 3-unsubstituted IZPs in the presence of Lewis acid. Yan et al., 2014 [51], developed a modified metal-free strategy employing TBHP as the oxidant and a TBAI catalyst in DMF (SR-7d). Bagdi et al., 2013 [52], established the synthesis of IZP and its derivatives using 2-aminopyridine and aryl ketones through C-H functionalization in the presence of a copper catalyst (SR-7e). At the same time, Chandra Mohan et al., 2013 [53], developed a synthesis method for IZP employing a CuI catalyst in a DMF solvent (SR-7f). Later, Cai et al., 2013 [54], demonstrated the synthesis of heteroaromatic IZPs in the presence of a copper iodide or boron trifluoride etherate catalyst (SR-7g). Another methodology was developed by Zhang et al., 2013 [55], for the synthesis of functionalized IZPs from aliphatic, aromatic, or unsaturated ketones in the presence of an In(OTf)_3_ catalyst (SR-7h).

In addition to these synthetic strategies, eco-friendly and straightforward procedures, like photochemical methods, have been applied for the synthesis of IZPs to overcome challenges like environmental issues and dependence upon non-renewable sources. Photochemical methods offer many advantages over traditional heating strategies, notably using visible light and benign organic catalysts and solvents. Photocatalytic reactions have been applied with metallic or metal-free catalysts for the synthesis and functionalization of IZPs. Tran et al., 2022, reviewed the literature on recent advancements in the photochemical synthesis of IZPs [56].

## 3. Antibacterial Profile of Imidazopyridines

IZP is one of the most important scaffolds amongst various fused heterocyclic systems. It possess a broad range of pharmacological activities. Although significant research has been carried out against bacterial infections, only one drug, rifaximin, is available as an antibiotic on the market. There is considerable evidence to support the antibacterial activity of IZP. This may pave the way for the development of antibiotics to overcome resistance. An evaluation of the antibacterial qualities of IZPs suggested the involvement of various pathways in their mechanism of action. IZPs can target several enzymes associated with the synthesis of cell wall/peptidoglycan, protein, folic acid, DNA, or RNA, to eradicate infections. Investigations have exemplified various substituents on IZP rings which afford significant antibacterial activity. Once again, IZP is a multitargeted scaffold.

**Figure 3:**
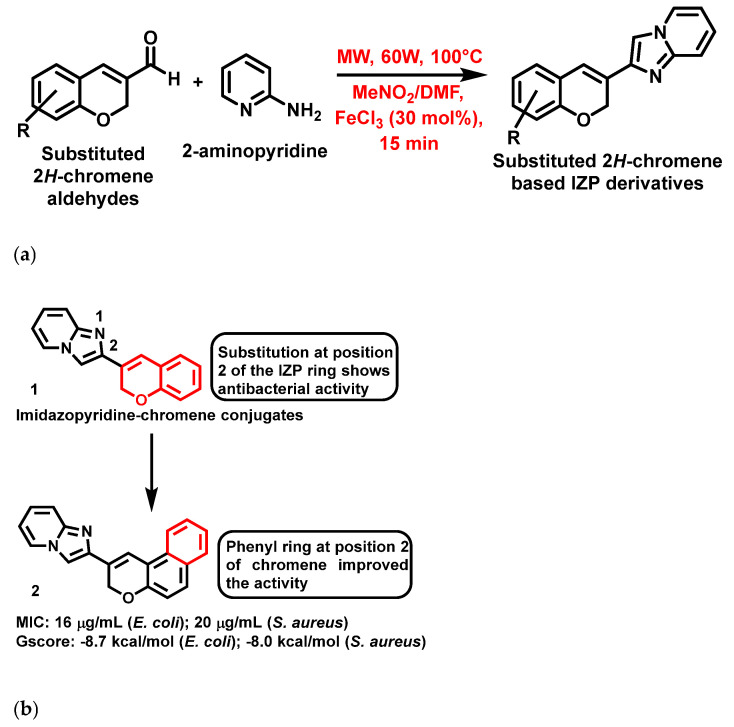
(**a**) General synthetic scheme of 2H-chromene based IZP derivatives. Signatures of compounds according to Mishra et al., 2021 [57]. (**b**) 2H-Chromene-based imidazopyridines with a brief SAR. Signatures of compounds according to Mishra et al., 2021 [57].

In 2021, Mishra et al. [57], reported that 2H-chromene-based IZP derivatives (**1**, Figure 3a,b) were potent peptide deformylase inhibitors. These compounds were synthesized using an eco-friendly, one-pot, three-component approach employing FeCl_3_ as the catalyst under microwave irradiation at 60W, 100 °C in 15 min (Figure 3a). In addition, these compounds exhibited potent antibacterial activity against bacterial strains such as *Klebsiella oxytoca*, *Streptococcus pyogenes*, *Staphylococcus aureus* (*S. aureus*) and *Escherichia coli* (*E. coli*). The most active compound is represented in Figure 3b, with its MIC and docking values.

**Figure 4:**
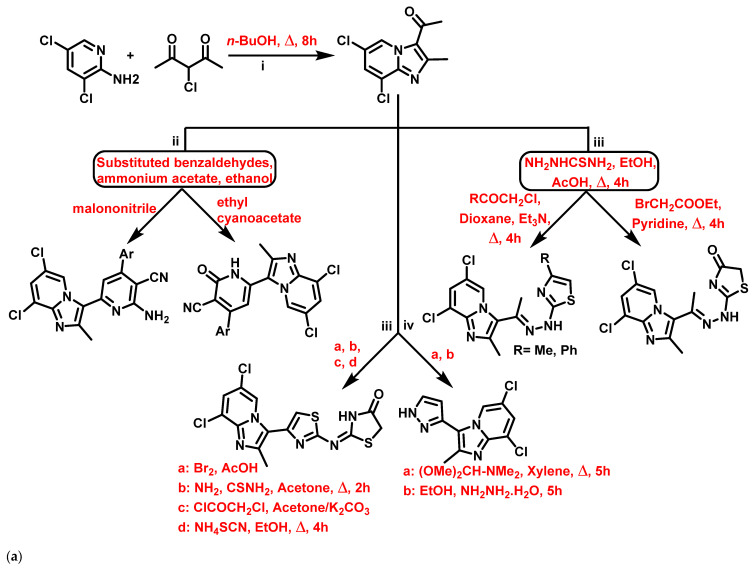

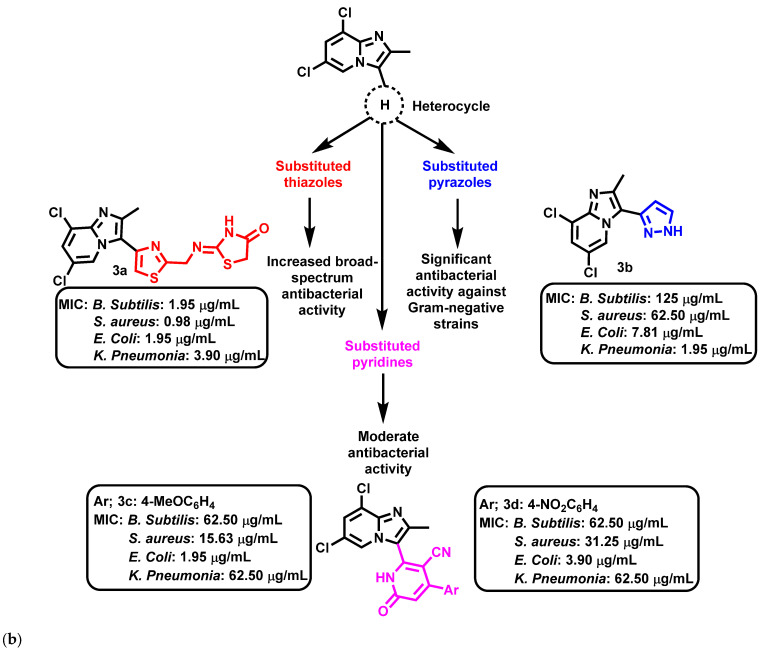
(**a**) Synthesis of (i) 3-acetyl-6,8-dichloro-2-methyl IZP, (ii) 6,8-dichloro-2-methylimidazo [1,2-a]pyridinyl-pyridine hybrids, (iii) 6,8-dichloro-2-methylimidazo [1,2-a]pyridinyl-thiazole hybrids, and (iv) 3-)pyrazolyl)-imidazo [1,2-a]pyridine hybrid. The signatures of these compounds according to Althagafi et al., 2021 [58]. (**b**) Imidazopyridine-based heterocycles, including pyridine-, pyrazole, and thiazole-substituted systems. Signatures of compounds according to Althagafi et al., 2021 [58].

Althagafi et al., 2021 [58], reported the synthesis of IZPs using substituted 2-APs and 3-chloroacetylacetone. The prepared IZPs reacted with substituted benzaldehyde and malononitrile or ethyl cyanoacetate in a three-component reaction. The synthetic schemes for the preparation of IZP and its derivatives are presented in Figure 3a. The IZP-based heterocycles (**3a**–**d**) were found to be more effective against *E. coli*, *S. aureus*, *Bacillus subtilis* (*B. subtilis*), *Klebsiella pneumonia* (*K. pneumonia*) than ampicillin and gentamicin. In summary, thiazole-based IZPs showed higher antibacterial activity than pyridine- or pyrazole-based IZPs against all tested organisms (Figure 4b). Thus, thiazole-based IZPs (**3a**) provide an opportunity in medicinal chemistry to find novel antibacterial agents.

**Figure 5:**
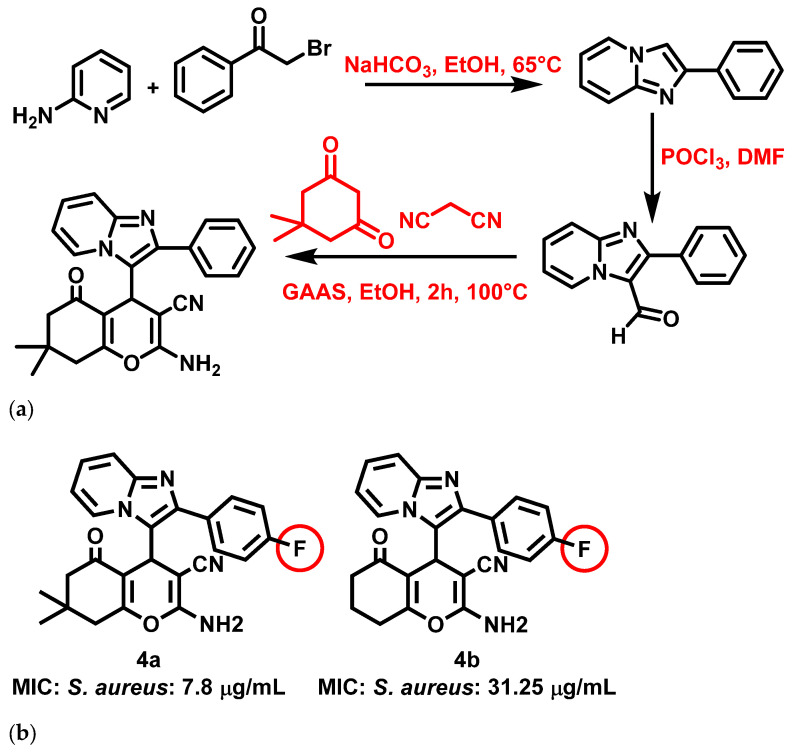
(**a**) Synthesis of 2-phenylimidazo [1,2-a] pyridine-based pyran bis-heterocycles. Signatures of compounds according to Thakur et al., 2020 [59]. (**b**) Imidazopyridine conjoined pyran bis-heterocyclic derivatives. Signatures of compounds according to Thakur et al., 2020 [59].

Thakur et al., 2020 [59], reported that IZP conjoined pyran bis-heterocyclic derivatives are potent antibacterial agents. The derivatives were synthesized by ultrasound-assisted synthetic reactions combining multicomponent reactions, an eco-friendly catalyst (gluconic acid aqueous solution), and a green solvent. The synthesis of an IZP-pyran heterocycle involved malononitrile, cyclochexanedione, and substituted IZPs. The synthetic reaction mechanism encompassed Knoevenagel condensation and Michael reaction, followed by cyclization and tautomerization (Figure 5a). Antibacterial studies showed significant results for the synthesized compounds against *E. coli*, *S. aureus*, and *Salmonella typhi* (*S. typhi*). Amongst the 11 synthesized derivatives, two molecules, i.e., **4a** and **4b**, exhibited particularly strong activity against *S. aureus* (**4a**: 7.8 μg/mL **4b**: 31.25 μg/mL) (Figure 5b). Thakur et al. noted that the presence of fluorine atoms at the para position of the phenyl ring of IZP increased the activity of these compounds against *S. aureus*. Further, the derivatives also yielded a pronounced (<10%) hemolytic effect, indicating that the compounds were benign to erythrocytes, as evidenced by hemolysis tests.

**Figure 6:**
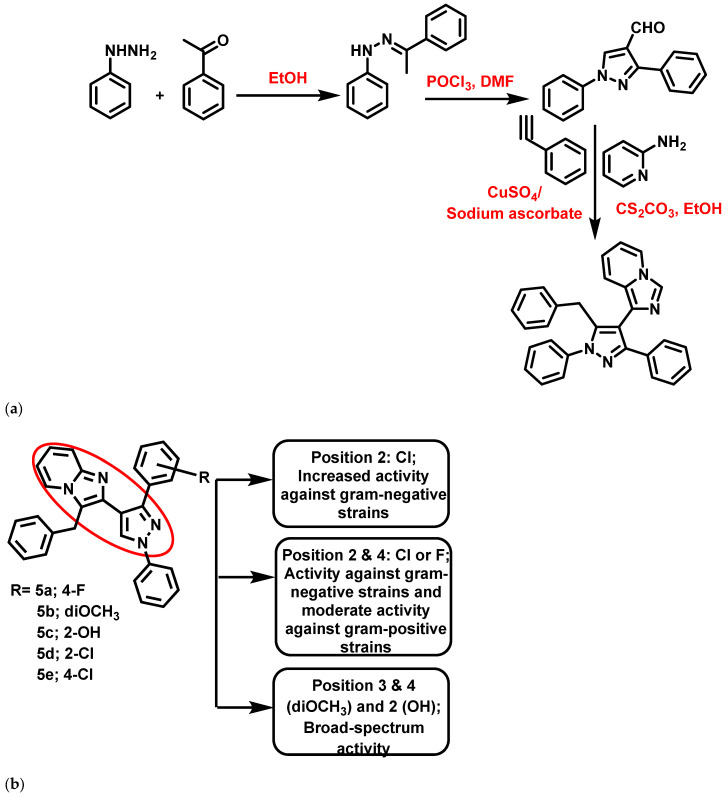
(**a**) Synthetic scheme of pyrazole-imidazo [1,2-a]pyridine derivatives. Signatures of compounds according to Ebenezer et al., 2019 [60]. (**b**) Pyrazolo-imidazopyridine molecular conjugates. Signatures of compounds according Ebenezer et al., 2019 [60].

In 2019, Ebenezer et al. [60] reported pyrazolo-IZP molecular conjugates as antibacterial agents targeting cell wall synthesis. These derivatives were synthesized using a one-pot, three-component tandem reaction employing CuSO_4_/Na-ascorbate and Cs_2_CO_3_ as a catalyst. Amongst the 12 synthesized derivatives, 5 (Figure 6) exhibited significant bactericidal activity (zone of inhibition >9 mm) against Methicillin-Resistant *Staphylococcus aureus* (MRSA), *S. aureus*, *E. coli*, *S. typhi*, *K. pneumonia*, *Pseudomonas aeruginosa* (*P. aeruginosa*) compared to ciprofloxacin. In addition, the conjugates were subjected to molecular docking analysis against glucosamine-6-phosphate (GlcN-6-P) synthase enzyme. From MBC studies, it was clear that compound **5a** exhibited broad-spectrum inhibitory activity, with MBC values <2.50 μg/mL against a selected panel of bacteria. In contrast, compounds **5b** and **5d** showed significant activity only against Gram-negative strains (<1 μg/mL), while compound **5c** (2-OH substituted) presented activity against both Gram-positive (*S. aureus*: 0.08 μg/mL; MRSA:19.53 μg/mL) and Gram-negative (*S. typhi*:0.63 μg/mL; *K. pneumonia*: 0.08 μg/mL; *P. aeruginosa*:0.63 μg/mL) strains. Compound **5e** also showed significant bactericidal properties (<1 μg/mL) against all of the tested strains. Supporting the in vitro data, a docking analysis revealed the significant binding affinity with the key amino acid residue of the catalytic pocket of the GlcN-6-P synthase enzyme.

**Figure 7:**
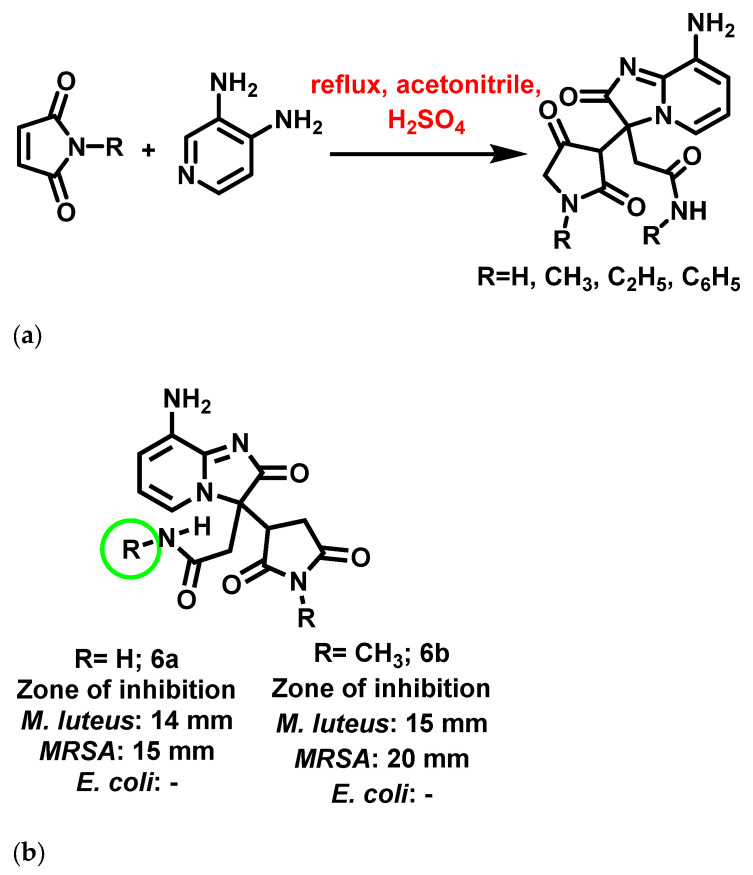
(**a**) Synthetic scheme of dihydroimidazo [1,2-a]pyridine derivatives. Signatures of compounds according to Salhi et al., 2019 [61]. (**b**) Most potent dihydroimidazopyridines in the study. Signatures of compounds according to Salhi et al., 2019 [61].

At the same time Salhi et al., 2019 [61], reported dihydro-IZPs as potential antibacterial agents against wild and resistant bacterial strains such as *E. coli*, *P. aeruginosa*, *S. aureus*, *Enterococcus faecalis* (*E. faecalis*), MRSA, and Methicillin-Resistant *Micrococcus luteus* (MRML). Salhi synthesized these compounds using maleimide as a starting material with kegging-type hetero polyacid (H_4_SiW_12_O_40_.nH_2_O) as a catalyst. The synthetic mechanism comprised a double condensation reaction of maleimide with APs, which acts as a nucleophile (Michael addition) and as a basic reagent (Knoevenagel reaction) (Figure 7a). Out of the four synthesized IZPs, two (Figure 7b) showed significant broad-spectrum bactericidal activity. SAR suggested that substitutions (R) of either hydrogen or methyl groups on the dihydropyrrole moiety and side chain enhanced the activity of **6a** and **6b**. In contrast, increasing the carbon chain to ethyl or phenyl groups showed detrimental effects on the aforementioned activity.

**Figure 8:**
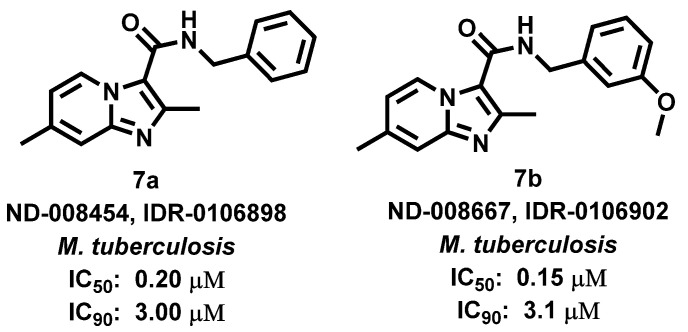
Imidazopyridine derivatives showing activity *M. tuberculosis.* Signatures of compounds according to Malley et al., 2018 [62].

Malley et al., 2018 [62], reported on the use of IZPs (Figure 8) as direct targets of QcrB, a component of terminal cytochrome oxidase which is involved in the electron transport chain of *Mycobacterium tuberculosis* (Mtb). As IZPs show chemical resemblance with the clinical candidate drug Telacebec (Q203, antitubercular drug), proposed to affect QcrB directly, Malley predicted that IZPs may also target QcrB. In conclusion, compounds **7a** and **7b** exhibited significant bactericidal properties against Mtb by disrupting pH homeostasis and depleting intracellular ATP levels.

**Figure 9:**
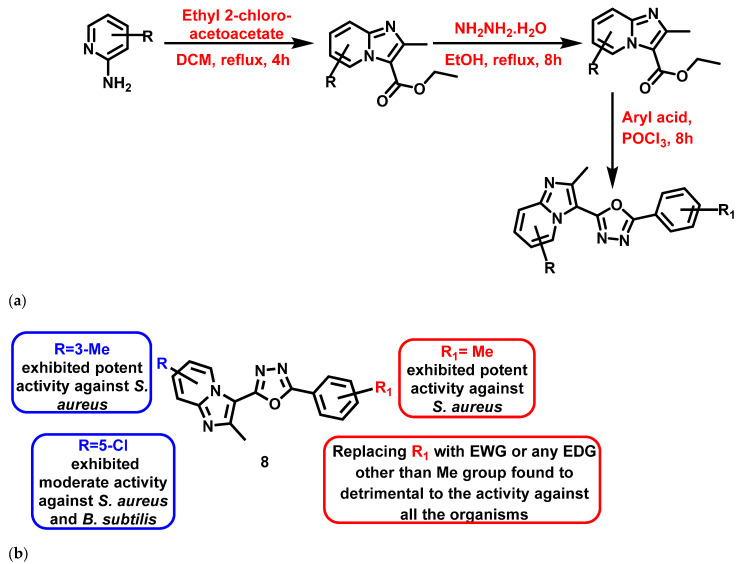
(**a**) Synthetic scheme of newly IZP derivatives flanged with oxadiazole nucleus. Signatures of compounds according to Kuthyala et al., 2018 [63]. (**b**) A brief SAR of trisubstituted imidazopyridine-oxadiazole hybrids. Signatures of compounds according to Kuthyala et al., 2018 [63].

Again in 2018, Kuthyala et al. [63] reported the synthesis and evaluated the antibacterial properties of trisubstituted IZPs. The derivatives were synthesized by engrossing oxadiazole in the presence of dichloromethane, hydrazine hydrate, ethanol, and POCl_3_ in different steps (Figure 9a). The compounds exhibited pronounced bactericidal activity, as evidenced by their MIC and time-kill kinetics. Out of 9 synthesized compounds with varying substitutions, compound (**8**) (Figure 9b), containing methyl and nitro groups on the IZP and phenyl rings, exhibited significant antibacterial activity (*S. aureus*: 3.12 µg/mL) compared to the reference compound, ciprofloxacin. The results are summarized in Figure 9.

**Figure 10:**
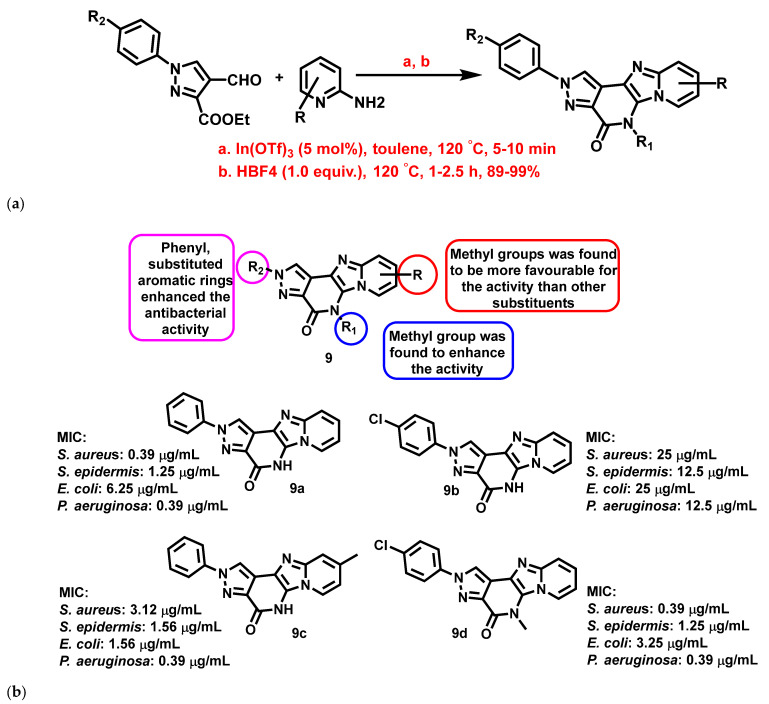
(**a**) Synthetic scheme of pyrazolopyridinone fused IZP derivatives. Signatures of compounds according to Devi et al., 2017 [64]. (**b**) A brief SAR of pyrazolopyridinone fused imidazopyridine derivatives. Signatures of compounds according to Devi et al., 2017 [64].

Devi et al., 2017 [64], synthesized pyrazolopyridinone-fused IZPs using one-pot tandem reactions through an In(OTf)-assisted Groebke-Blackburn Bienayme multi-component strategy (Figure 10a). The compounds exhibited significant bactericidal activity against *E. coli*, *P. aeruginosa*, *S. aureus*, and *Staphylococcus epidermis* (*S. epidermis*) when compared with ofloxacin. A few highly active compounds (**9a**–**d**) with different substitutions are represented in Figure 10. A preliminary SAR analysis (**9**, Figure 10b) revealed that substitution on the nitrogen of the pyrazole moiety with aryl or aryl substituted groups enhanced the antibacterial activity of the compounds.

**Figure 11:**
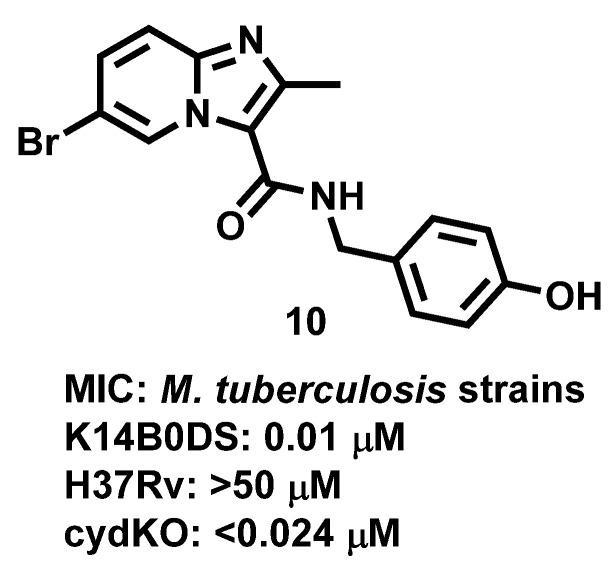
Imidazopyridine derivative exhibiting activity against different strains of *M. tuberculosis.* Signatures of compounds according to Arora et al., 2014 [65].

Further, Arora et al., 2014 [65], proposed that IZP (**10**) derivatives are potent antiMtb compounds that were shown to target respiratory bc1 complex in laboratory-adapted strains of Mtb such as K14B0DS, H37Rv, cydKO. The results (Figure 11) highlighted the promiscuity of the bc1 complex in Mtb and the risk of targeting ATP or energy metabolism with novel therapeutics.

**Figure 12:**
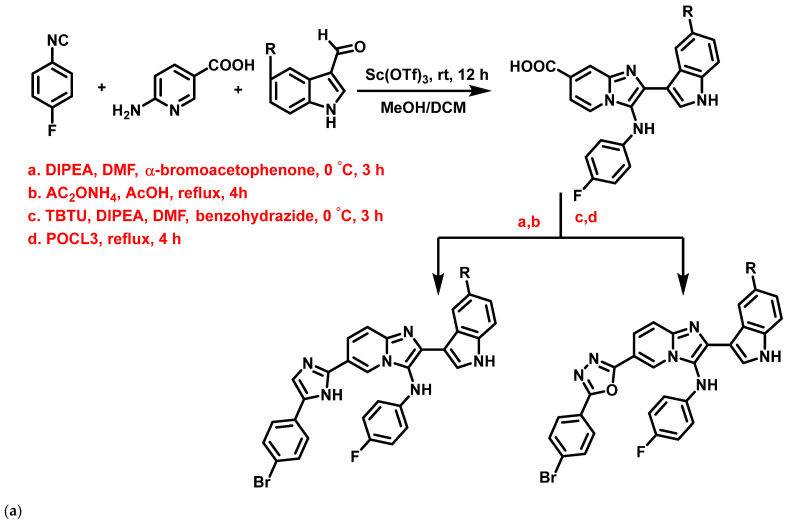

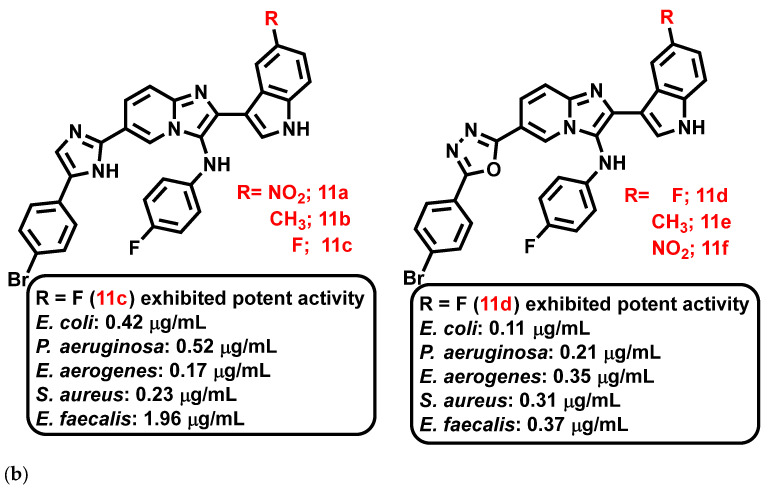
(**a**) General scheme for the synthesis of novel indole-based imidazopyridine derivatives. Signatures of compounds according to Al-Tel et al., 2011 [66]. (**b**) Indole-based imidazopyridine derivative exhibiting activity against different selected strains. Signatures of compounds according to Al-Tel et al., 2011 [66].

Al-Tel et al., 2011 [66], proposed indole based-IZPs as antibacterial agents, synthesized using the Groebke-Blackburn three-component reaction and Ugi reaction [4+1] cycloaddition protocol involving amino-azole, aldehyde, and an isocyanide with TBTU, DIPEA, DMF, DIPEA as catalysts (Figure 12a). These derivatives exerted strong inhibition against *S. aureus*, *E. faecalis*, *Bacillus megaterium* (*B. megaterium*), *E. coli*, *P. aeruginosa,* and *Enterococcus aerogenes* (*E. aerogenes*), in the range of 0.11–23.45 μg/mL, compared to control antibiotics such as cefixime and amoxicillin. In summary, the nature of substituents such as armed aryl groups exemplified the extent of the activity of these compounds (**11a–f**, Figure 12b). Preliminary SAR studies revealed that bromo-fluoro substituents enhanced the antibacterial activity significantly, compared to other substituents.

**Figure 13:**
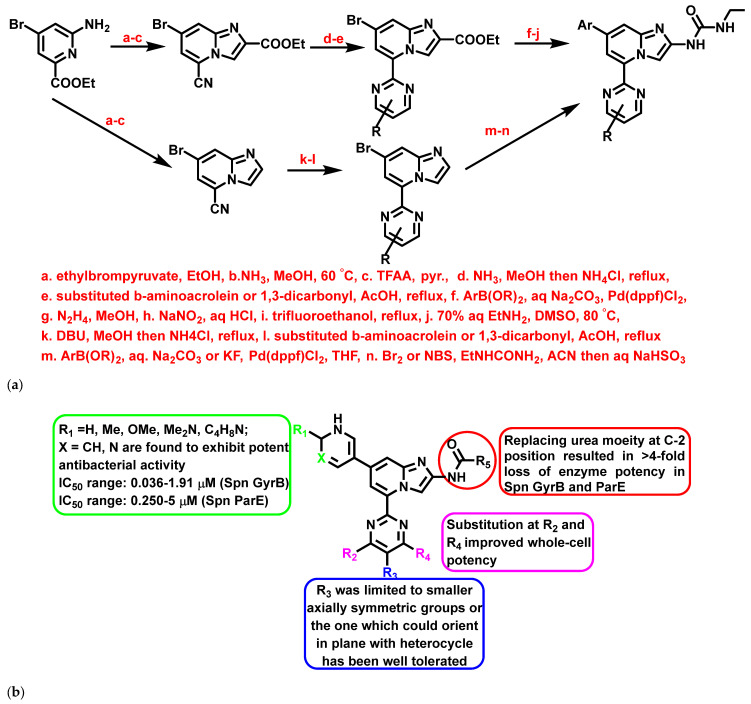
(**a**) General synthetic scheme of 5-(2-Pyrimidinyl)-imidazo [1,2-a]pyridine derivatives. Signatures of compounds according to Starr et al., 2009 [67]. (**b**) A brief SAR on imidazopyridine derivatives exemplifying the enzyme inhibitory activity. Signatures of compounds according to Starr et al., 2009 [67].

A study by Starr et al., 2009 [67], exemplified IZPs as antibacterial agents targeting, e.g., DNA gyrase and topoisomerase IV. The compounds were synthesized employing 2-amino-4-bromo-6-ethoxycarbonylpyridine as the starting material and using various reagents and catalysts. The reaction mechanism included the Suzuki coupling reaction and one-pot procedure to obtain greater yields (Figure 13a). The derivatives were found to be effective against *S. aureus*, MRSA, *S. pyogenes*, *S. pneumonia*, and Fluoroquinolone-Resistant *Streptococcus pneumonia* (FRSP), with significant MIC. In addition, a few derivatives exhibited significant enzyme inhibition activity against *S. pneumonia* GyrB (Spn GyrB) and ParE (Spn ParE) (Figure 13b). Further, Starr et al. also performed in vivo and pharmacokinetic studies for the most potent compounds. The results were stunning: pyridyl-substituted IZPs established excellent oral PD_50_ and PK parameters in murine *S. pyogenes* sepsis and *S. pneumonia* lung models.

## 4. Conclusions

Imidazopyridine (IZP)-based derivatives have a broad range of pharmacological activities. Some IZP-based conjugates have been extensively utilized for the treatment of bacterial infections caused by wild or resistant bacteria. The intriguing scaffold of IZP plays a key role in the advancement of novel antibiotics. Because of its immense pharmacological significance, many synthetic approaches have been developed to apply diverse substitutions to this scaffold. In recent years, IZPs have been synthesized and screened for their in vitro and in vivo activity against Gram-negative, Gram-positive, and resistant strains of bacteria. This review highlights the synthetic strategies to develop IZPs and ongoing advances in their potential antibacterial activity against wild and mutant strains of bacterial pathogens. This information might be used to develop new and diverse IZP derivatives to combat infections and resistance.

## Data Availability

Not applicable.

## References

[1] Leeson P.D., Springthorpe B. (2007). The influence of drug-like concepts on decision-making in medicinal chemistry. Nat. Rev. Drug Discov..

[2] Labarrios E.M., Delgado F., Tamariz J., Aguilar R.l. (2015). Synthesis of Î±-ketols by functionalization of captodative alkenes and divergent preparation of heterocycles and natural products. Tetrahedron.

[3] Kerru N., Gummidi L., Maddila S., Gangu K.K., Jonnalagadda S.B. (2020). A review on recent advances in nitrogen-containing molecules and their biological applications. Molecules.

[4] Ju Y., Varma R.S. (2006). Aqueous N-heterocyclization of primary amines and hydrazines with dihalides: Microwave-assisted syntheses of N-azacycloalkanes, isoindole, pyrazole, pyrazolidine, and phthalazine derivatives. J. Org. Chem..

[5] Eftekhari-Sis B., Zirak M., Akbari A. (2013). Arylglyoxals in synthesis of heterocyclic compounds. Chem. Rev..

[6] Couty F., Evano G., Katritzky A., Ramsden C., Scriven E., Taylor R., Katritzky A.R., Ramsden C.A., Scriven E.F.V., Taylor R.J.K. (2008). Comprehensive Heterocyclic Chemistry III.

[7] Enguehard-Gueiffier C., Gueiffier A. (2007). Recent Progress in the Pharmacology of Imidazo [1, 2-a] pyridines. Mini Rev. Med. Chem..

[8] Kishbaugh T.L.S. (2016). Pyridines and Imidazopyridines with medicinal significance. Curr. Top. Med. Chem..

[9] Abignente E. (1991). Etudes d’imidazo [1, 2-a] pyridines et d’analogues douées d’activité anti-inflammatoire. Actual. Chim. Thér..

[10] Rival Y., Grassy G., Michel G. (1992). Synthesis and antibacterial activity of some imidazo[1,2-a]pyrimidine derivatives. Chem. Pharm. Bull..

[11] Hamdouchi C., de Blas J., del Prado M., Gruber J., Heinz B.A., Vance L. (1999). 2-Amino-3-substituted-6-[(E)-1-phenyl-2-(*N*-methylcarbamoyl) vinyl] imidazo [1, 2-a] pyridines as a novel class of inhibitors of human rhinovirus: Stereospecific synthesis and antiviral activity. J. Med. Chem..

[12] Rupert K.C., Henry J.R., Dodd J.H., Wadsworth S.A., Cavender D.E., Olini G.C., Fahmy B., Siekierka J.J. (2003). Imidazopyrimidines, potent inhibitors of p38 MAP kinase. Bioorg. Med. Chem. Lett..

[13] Hranjec M., Kralj M., Piantanida I., Sedić M., Šuman L., Pavelić K., Karminski-Zamola G. (2007). Novel cyano-and amidino-substituted derivatives of styryl-2-benzimidazoles and benzimidazo [1, 2-a] quinolines. Synthesis, photochemical synthesis, DNA binding, and antitumor evaluation, part 3. J. Med. Chem..

[14] Kotovskaya S., Baskakova Z., Charushin V., Chupakhin O., Belanov E., Bormotov N., Balakhnin S., Serova O. (2005). Synthesis and antiviral activity of fluorinated pyrido [1, 2-a] benzimidazoles. Pharm. Chem. J..

[15] Lhassani M., Chavignon O., Chezal J.-M., Teulade J.-C., Chapat J.-P., Snoeck R., Andrei G., Balzarini J., De Clercq E., Gueiffier A. (1999). Synthesis and antiviral activity of imidazo [1, 2-a] pyridines. Eur. J. Med. Chem..

[16] Boerner R., Moller H. (1997). Saripidem-a new treatment for panic disorders. Psychopharmakotherapie.

[17] Yukiiri K., Mizushige K., Ueda T., Nishiyama Y., Aoyama T., Kohno M. (2001). Effects of olprinone, a phosphodiesterase 3 inhibitor, on regional cerebral blood flow of cerebral cortex in stroke patients. J. Cardiovasc. Pharmacol..

[18] Langer S., Arbilla S., Benavides J., Scatton B. (1990). Zolpidem and alpidem: Two imidazopyridines with selectivity for omega 1-and omega 3-receptor subtypes. Adv. Biochem. Psychopharmacol..

[19] Almirante L., Polo L., Mugnaini A., Provinciali E., Rugarli P., Biancotti A., Gamba A., Murmann W. (1965). Derivatives of imidazole. I. Synthesis and reactions of imidazo [1, 2-α] pyridines with analgesic, antiinflammatory, antipyretic, and anticonvulsant activity. J. Med. Chem..

[20] Koo H.L., DuPont H.L. (2010). Rifaximin: A unique gastrointestinal-selective antibiotic for enteric diseases. Curr. Opin. Gastroenterol..

[21] Ponnala S., Kiran Kumar S., Bhat B.A., Prasad Sahu D. (2005). Synthesis of bridgehead nitrogen heterocycles on a solid surface. Synth. Commun..

[22] Zhu D.-J., Chen J.-X., Liu M.-C., Ding J.-C., Wu H.-Y. (2009). Catalyst: And solvent-free synthesis of imidazo [1, 2-a] pyridines. J. Brazil. Chem. Soc..

[23] Stasyuk A.J., Banasiewicz M., Cyrański M.K., Gryko D.T. (2012). Imidazo [1, 2-a] pyridines susceptible to excited state intramolecular proton transfer: One-pot synthesis via an Ortoleva–King reaction. J. Org. Chem..

[24] Yadav J., Reddy B.S., Rao Y.G., Srinivas M., Narsaiah A. (2007). Cu (OTf) 2-catalyzed synthesis of imidazo [1, 2-a] pyridines from α-diazoketones and 2-aminopyridines. Tetrahedron Lett..

[25] Xie Y.-Y., Chen Z.-C., Zheng Q.-G. (2002). Organic reactions in ionic liquids: Ionic liquid-accelerated cyclocondensation of α-tosyloxyketones with 2-aminopyridine. Synthesis.

[26] Ueno M., Togo H. (2004). Environmentally benign preparation of heteroaromatics from ketones or alcohols, with macroporous polystyrenesulfonic acid and (diacetoxyiodo) benzene, followed by thioamide, amidine, and 2-aminopyridine. Synthesis.

[27] Liu Z., Chen Z.-C., Zheng Q.-G. (2004). Hypervalent iodine in synthesis. 94. A facile synthesis of 2-substituted-imidazo [1, 2-a] pyridines by cyclocondensation of alkynyl (phenyl) iodonium salts and 2-aminopyridine. Synth. Commun..

[28] Wu Z., Pan Y., Zhou X. (2011). Synthesis of 3-arylimidazo [1, 2-a] pyridines by a catalyst-free cascade process. Synthesis.

[29] Yu C., Chen X., Wu R., Yang G., Shi J., Pan L. (2014). One-Pot Synthesis of N-(Imidazo [1, 2-a] pyridin-3-yl)-Substituted Sulfonamides Using Catalytic Zinc Chloride. Eur. J. Org. Chem..

[30] Nair D.K., Mobin S.M., Namboothiri I.N. (2012). Synthesis of imidazopyridines from the Morita–Baylis–Hillman acetates of nitroalkenes and convenient access to Alpidem and Zolpidem. Org. Lett..

[31] Yan H., Yang S., Gao X., Zhou K., Ma C., Yan R., Huang G. (2012). Iron (II)-catalyzed denitration reaction: Synthesis of 3-methyl-2-arylimidazo [1, 2-a] pyridine derivatives from aminopyridines and 2-methylnitroolefins. Synlett.

[32] Santra S., Bagdi A.K., Majee A., Hajra A. (2013). Iron (III)-Catalyzed Cascade Reaction between Nitroolefins and 2-Aminopyridines: Synthesis of Imidazo [1, 2-a] Pyridines and Easy Access towards Zolimidine. Adv. Synth. Catal..

[33] Yan H., Wang Y., Pan C., Zhang H., Yang S., Ren X., Li J., Huang G. (2014). Iron(III)-Catalyzed Denitration Reaction: One-Pot Three-Component Synthesis of Imidazo[1,2-a]pyridine Derivatives. Eur. J. Org. Chem..

[34] Schwerkoske J., Masquelin T., Perun T., Hulme C. (2005). New multi-component reaction accessing 3-aminoimidazo [1, 2-a] pyridines. Tetrahedron Lett..

[35] DiMauro E.F., Kennedy J.M. (2007). Rapid synthesis of 3-amino-imidazopyridines by a microwave-assisted four-component coupling in one pot. J. Org. Chem..

[36] Adib M., Sheibani E., Zhu L.-G., Mirzaei P. (2008). An efficient synthesis of 3-amino-2-arylimidazo [1, 2-a] pyridines. Tetrahedron Lett..

[37] Shao N., Pang G.-X., Yan C.-X., Shi G.-F., Cheng Y. (2011). Reaction of β-lactam carbenes with 2-pyridyl isonitriles: A one-pot synthesis of 2-carbonyl-3-(pyridylamino) imidazo [1, 2-a] pyridines useful as fluorescent probes for mercury ion. J. Org. Chem..

[38] Khan A.T., Basha R.S., Lal M. (2012). Bromodimethylsulfonium bromide (BDMS) catalyzed synthesis of imidazo [1, 2-a] pyridine derivatives and their fluorescence properties. Tetrahedron Lett..

[39] Ramesha A.B., Raghavendra G.M., Nandeesh K.N., Rangappa K.S., Mantelingu K. (2013). Tandem approach for the synthesis of imidazo [1, 2-a] pyridines from alcohols. Tetrahedron Lett..

[40] Chernyak N., Gevorgyan V. (2010). General and Efficient Copper-Catalyzed Three-Component Coupling Reaction towards Imidazoheterocycles: One-Pot Synthesis of Alpidem and Zolpidem. Angew. Chem..

[41] Yu J., Jin Y., Zhang H., Yang X., Fu H. (2013). Copper-Catalyzed Aerobic Oxidative C-H Functionalization of Substituted Pyridines: Synthesis of Imidazopyridine Derivatives. Chem. Eur. J..

[42] Huang H., Ji X., Tang X., Zhang M., Li X., Jiang H. (2013). Conversion of pyridine to imidazo [1, 2-a] pyridines by copper-catalyzed aerobic dehydrogenative cyclization with oxime esters. Org. Lett..

[43] Monir K., Kumar Bagdi A., Mishra S., Majee A., Hajra A. (2014). Copper (II)-Catalyzed Aerobic Oxidative Coupling between Chalcone and 2-Aminopyridine via C-H Amination: An Expedient Synthesis of 3-Aroylimidazo [1, 2-a] pyridines. Adv. Synth. Catal..

[44] Wang H., Wang Y., Peng C., Zhang J., Zhu Q. (2010). A direct intramolecular C− H amination reaction cocatalyzed by copper (II) and iron (III) as part of an efficient route for the synthesis of pyrido [1, 2-a] benzimidazoles from *N*-aryl-2-aminopyridines. J. Am. Chem. Soc..

[45] Masters K.S., Rauws T.R., Yadav A.K., Herrebout W.A., Van der Veken B., Maes B.U. (2011). On the importance of an acid additive in the synthesis of pyrido [1, 2-a] benzimidazoles by direct copper-catalyzed amination. Chem. Eur. J..

[46] Wang H., Wang Y., Liang D., Liu L., Zhang J., Zhu Q. (2011). Copper-Catalyzed Intramolecular Dehydrogenative Aminooxygenation: Direct Access to Formyl-Substituted Aromatic N-Heterocycles. Angewandte Chem..

[47] Chioua M., Soriano E., Infantes L., Jimeno M.L., Marco-Contelles J., Samadi A. (2013). Silver-Catalyzed Cyclization of N-(Prop-2-yn-1-yl) pyridin-2-amines. Eur. J. Org. Chem..

[48] Donohoe T.J., Kabeshov M.A., Rathi A.H., Smith I.E. (2012). Direct preparation of thiazoles, imidazoles, imidazopyridines and thiazolidines from alkenes. Org. Biomol. Chem..

[49] Zeng J., Tan Y.J., Leow M.L., Liu X.-W. (2012). Copper (II)/iron (III) Co-catalyzed intermolecular diamination of alkynes: Facile synthesis of imidazopyridines. Org. Lett..

[50] Gao Y., Yin M., Wu W., Huang H., Jiang H. (2013). Copper-Catalyzed Intermolecular Oxidative Cyclization of Halo-alkynes: Synthesis of 2-Halo-substituted Imidazo [1, 2-a] pyridines, Imidazo [1, 2-a] pyrazines and Imidazo [1, 2-a] pyrimidines. Adv. Synth. Catal..

[51] Yan R.-L., Yan H., Ma C., Ren Z.-Y., Gao X.-A., Huang G.-S., Liang Y.-M. (2012). Cu (I)-catalyzed synthesis of imidazo [1, 2-a] pyridines from aminopyridines and nitroolefins using air as the oxidant. J. Org. Chem..

[52] Bagdi A.K., Rahman M., Santra S., Majee A., Hajra A. (2013). Copper-Catalyzed Synthesis of Imidazo [1, 2-a] Pyridines through Tandem Imine Formation-Oxidative Cyclization under Ambient Air: One-Step Synthesis of Zolimidine on a Gram-Scale. Adv. Synth. Catal..

[53] Chandra Mohan D., Reddy Donthiri R., Nageswara Rao S., Adimurthy S. (2013). Copper (I) Iodide-Catalysed Aerobic Oxidative Synthesis of Imidazo [1, 2-a] Pyridines from 2-Aminopyridines and Methyl Ketones. Adv. Synth. Catal..

[54] Cai Z.J., Wang S.Y., Ji S.J. (2013). Copper (I) Iodide/Boron Trifluoride Etherate-Cocatalyzed Aerobic Dehydrogenative Reactions Applied in the Synthesis of Substituted Heteroaromatic Imidazo [1, 2-a] Pyridines. Adv. Synth. Catal..

[55] Zhang Y., Chen Z., Wu W., Zhang Y., Su W. (2013). CuI-catalyzed aerobic oxidative α-aminaton cyclization of ketones to access aryl or alkenyl-substituted imidazoheterocycles. J. Org. Chem..

[56] Tran C., Hamze A. (2022). Recent Developments in the Photochemical Synthesis of Functionalized Imidazopyridines. Molecules.

[57] Mishra N.P., Mohapatra S., Sahoo C.R., Raiguru B.P., Nayak S., Jena S., Padhy R.N. (2021). Design, one-pot synthesis, molecular docking study, and antibacterial evaluation of novel 2H-chromene based imidazo [1, 2-a] pyridine derivatives as potent peptide deformylase inhibitors. J. Mol. Struct..

[58] Althagafi I., Abdel-Latif E. (2021). Synthesis and antibacterial activity of new imidazo [1, 2-a] pyridines festooned with pyridine, thiazole or pyrazole moiety. Polycycl. Aromat. Comp..

[59] Thakur A., Pereira G., Patel C., Chauhan V., Dhaked R.K., Sharma A. (2020). Design, one-pot green synthesis and antimicrobial evaluation of novel imidazopyridine bearing pyran bis-heterocycles. J. Mol. Struct..

[60] Ebenezer O., Awolade P., Koorbanally N., Singh P. (2020). New library of pyrazole–imidazo [1, 2-α] pyridine molecular conjugates: Synthesis, antibacterial activity and molecular docking studies. Chem. Biol. Drug Des..

[61] Salhi L., Achouche-Bouzroura S., Nechak R., Nedjar-Kolli B., Rabia C., Merazig H., Poulain-Martini S., Dunach E. (2020). Synthesis of functionalized dihydroimidazo [1, 2-A] pyridines and 4-thiazolidinone derivatives from maleimide, as new class of antimicrobial agents. Synth. Commun..

[62] O’malley T., Alling T., Early J.V., Wescott H.A., Kumar A., Moraski G.C., Miller M.J., Masquelin T., Hipskind P.A., Parish T. (2018). Imidazopyridine compounds inhibit mycobacterial growth by depleting ATP levels. Antimicrob. Agents Chemother..

[63] Kuthyala S., Shankar M.K., Nagaraja G.K. (2018). Synthesis, Single-Crystal X-Ray, Hirshfeld and Antimicrobial Evaluation of some New Imidazopyridine Nucleus Incorporated with Oxadiazole Scaffold. Chem. Select.

[64] Devi N., Jana A.K., Singh V. (2018). Assessment of novel pyrazolopyridinone fused imidazopyridines as potential antimicrobial agents. Karbala Int. J. Mod. Sci..

[65] Arora K., Ochoa-Montaño B., Tsang P.S., Blundell T.L., Dawes S.S., Mizrahi V., Bayliss T., Mackenzie C.J., Cleghorn L.A., Ray P.C. (2014). Respiratory flexibility in response to inhibition of cytochrome C oxidase in Mycobacterium tuberculosis. Antimicrob. Agents Chemother..

[66] Al-Tel T.H., Al-Qawasmeh R.A., Zaarour R. (2011). Design, synthesis and in vitro antimicrobial evaluation of novel Imidazo [1, 2-a] pyridine and imidazo [2, 1-b][1, 3] benzothiazole motifs. Eur. J. Med. Chem..

[67] Starr J.T., Sciotti R.J., Hanna D.L., Huband M.D., Mullins L.M., Cai H., Gage J.W., Lockard M., Rauckhorst M.R., Owen R.M. (2009). 5-(2-Pyrimidinyl)-imidazo [1, 2-a] pyridines are antibacterial agents targeting the ATPase domains of DNA gyrase and topoisomerase IV. Bioorg. Med. Chem. Lett..

